# Bioengineering of Hair Follicle-like Structure for Validation of Hair Growth Promoting Compounds

**DOI:** 10.3390/bioengineering9110645

**Published:** 2022-11-03

**Authors:** Hyun Woo Joo, Min Kyu Kim, Soon Sun Bak, Young Kwan Sung

**Affiliations:** 1 Department of Immunology, School of Medicine, Kyungpook National University, Daegu 41944, Korea; 2 BK21 FOUR KNU Convergence Educational Program of Biomedical Sciences for Creative Future Talents, Department of Biomedical Sciences, School of Medicine, Kyungpook National University, Daegu 41944, Korea

**Keywords:** alopecia, hair follicles, hair loss, keratinocytes, screening, spheroids

## Abstract

We aimed to establish screening and efficacy test techniques for use in the development of hair-promoting agents. To this end, we used the dermal papilla cell (DPc)-derived immortalized cell line (SV40T-hTERT DPc) and neonatal foreskin-derived keratinocyte cell line (Ker-CT) to form an immortalized cell-based hair follicle-like structure. The SV40T-hTERT DPc spheroids exhibited a higher cell ratio in the spheroids than primary DPc spheroids, and SV40T-hTERT DPc aggregated with spheroids larger in diameter than primary DPc when the same cell number was seeded into the low-adhesion plate. Microscopic imaging and fluorescence staining results indicated that both primary and immortalized cell combinations form a hair follicle-like structure with a long-stretched keratinocyte layer under the condition that the spheroids have the same diameter as that of in vivo dermal papillary tissue in the hair follicle. The hair follicle-like structure elongation was increased upon treatment with three known hair follicle growth-promoting compounds (minoxidil, tofacitinib, and ascorbic acid) compared with that in the control group. Therefore, using immortalized cells to generate a coherent follicle-like structure, we have developed models for screening and evaluating hair-care materials commonly used in the industry.

## 1. Introduction

The hair follicle is a skin appendage made up of several cells, including stem cells. Hair follicles have a cyclic growth pattern divided into telogen, anagen, and catagen phases [1]. Owing to the unique characteristics of hair follicles, studies have been focused on regulating the stem cell-like capability of hair follicle-derived cells that induce hair follicles through a process termed “trichogenecity” to utilize them for hair follicle regeneration [2,3,4].

Hair loss or alopecia is a common condition that poses a psychological, social, and economic burden, thereby hindering the quality of life [5]. Therefore, several studies are being conducted to treat and prevent hair loss, including the development of drugs and cosmetics [6,7,8,9]. Owing to the increased medical and cosmetic demands in hair care, several studies are being conducted to screen for candidate therapies to promote hair growth or prevent hair loss [10]. However, the 2D-cultured cells traditionally used for cell-based screening assays are physiologically different from in vivo cells [11]. These differences cause inaccurate screening results, leading to poor efficiency in evaluating candidate treatments. Therefore, an alternative approach that does not rely on 2D-cultured cells is required to identify more effective candidates. The ethical issues related to animal experiments for cosmetic development preclude the use of animals for testing the safety and efficiency of new therapeutics. Furthermore, the use of human hair follicle cultures as an alternative for efficiency testing [12] is limited by its procedural complexity in acquiring hair follicles. Recently, stem cell organoids and multicellular spheroids have been used to construct hair follicle structure [13,14,15]. A recent paper reported methods for screening hair-promoting agents using multicellular spheroids derived from primary-cultured hair follicle cells [16]. However, this method cannot be applied in the industry owing to differences in drug sensitivity among donors and loss of hair follicle formation ability and characteristics during continuous 2D culture of hair follicle cells. Therefore, a new type of cell-based anti-alopecia screening method must be developed.

**Figure 1:**
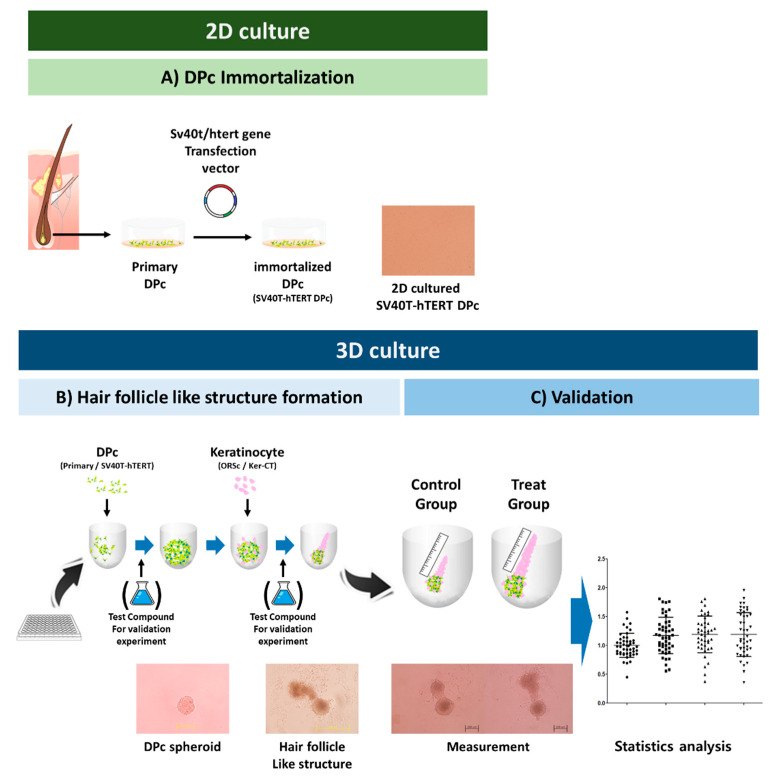
Schematic workflow. (**A**) Human hair follicle-derived cells (DPc, ORSc) were isolated and used in passages 2–3. The SV40T-hTERT DPc, an immortalized dermal papilla cell line, was previously described [17]. (**B**) 3D culture formation for constructing hair- follicle-like structure. (**C**) Quantitative analysis for the validation of the hair-promoting efficiency of the test substances. The elongated keratinocyte layer as observed using a microscope. The quantification obtained using the ImageJ software is presented as dot plots. The different symbols represent different concentrations of test substances.

In this study, we developed an immortalized cell-only hair follicle-like structure (iHFLS) using an immortalized human dermal papilla cell line previously developed by our group [17], and a commercial immortalized human foreskin keratinocyte cell line. We further monitored their characteristics and the extent to which they could recapitulate the hair physiology. Finally, we applied hair-promoting agents to our iHFLS and investigated whether they could promote the elongation of the keratinocyte layer (Figure 1). We believe our results would provide a novel immortalized multicellular hair follicle-like structure-based validation model for screening hair loss therapeutics and accelerate the search for new and effective hair-care compounds.

## 2. Materials and Methods

### 2.1. Cell Culture

Human hair follicle tissues in the form of residual hair were provided by the Hair Transplantation Center of Kyungpook National University after autologous transplantation surgery with consent from the patients. Human dermal papilla cells (DPc) were isolated from the bulbs of dissected hair follicles (HFs) and transferred onto plastic dishes, and cultured in Dulbecco’s modified Eagle medium (DMEM; Hyclone, Logan, UT, USA) supplemented with penicillin–streptomycin (Gibco, Burlington, ON, Canada), and 20% heat-inactivated fetal bovine serum (FBS; Hyclone, Logan, UT, USA), at 37 °C with 5% CO_2_. After cell outgrowth to the attached dermal papilla, DPc were incubated for 18 days, with the medium changed every three days. DPc were harvested with 0.25% trypsin/10 mM EDTA (Gibco, Burlington, ON, Canada) and sub-cultured at a split ratio of 1:3. Subsequently, DPc were maintained in DMEM with penicillin–streptomycin and 10% FBS.

Hair keratinocyte outer root sheath cells (ORSc) were isolated from the hair in which a hair shaft and hair bulb region is amputated. Trimmed hair were placed in a collagen type 1-coated plate with DMEM containing 20% FBS. The trimmed hair attached to the plate within two days; subsequently, the medium was changed to EpiLife medium (Gibco BRL, Gaithersburg, MD, USA) containing 1% antibiotic–antimycotic solution (Gibco, Burlington, ON, Canada) and 1% EpiLife defined growth supplement (EDGS; Gibco BRL, Gaithersburg, MD, USA). ORSc were incubated at 37 °C with 5% CO_2_. Primary cells from passages 2–3 were used in this study.

The immortalized DP cell line SV40T-hTERT DPc, developed by our research group were used in this study [17]. SV40T-hTERT DPc proliferate beyond passage 160 without showing senescence and supply a sufficient number of cells resulting in more reproducible in vitro results. SV40T-hTERT DPc were incubated in DMEM with penicillin–streptomycin and 10% FBS at 37 °C with 5% CO_2_.

Immortalized keratinocyte cells (Ker-CT) were purchased from ATCC, Manassas, VA, USA. Ker-CT cells were cultured in KGM-Gold BulletKit medium (Lonza, Basel, Switzerland) as recommended by the manufacturer.

### 2.2. Formation and Elongation of Hair Follicle-like Structure

For the spheroid formation, DPc were harvested with 0.25% trypsin/10 mM EDTA in diluted culture medium. Approximately 100 μL of the DPc suspension containing 300–3000 cells were seeded into each well of a 96-well hydrowell plate. The plates were centrifuged (250× *g*) and incubated for over 24 h until spheres were formed. Subsequently, the spheroid diameters were measured.

After the DPc for making spheres were seeded, keratinocytes (ORSc or Ker-CT) were trypsinized and suspended in three types of culture media: DMEM, KGM-Gold BulletKit medium, and William’s E media (Gibco, Burlington, ON, Canada), supplemented with 2 mM L-L-glutamine (Gibco, Burlington, ON, Canada), 100 U/mL penicillin–streptomycin, 10 ng/mL hydrocortisone (Sigma–Aldrich, Saint Louis, MO, USA), and 10 µg/mL insulin (Sigma–Aldrich, Saint Louis, MO, USA). Approximately 100 μL of the keratinocyte suspension containing 1000–3000 cells were added on the DPc in each well to develop hair follicle-like structures. The plate was then incubated for five days at 37 °C with 5% CO_2_, with the culture media changed every third day from that of incubation. For the validation of the hair-promoting agent, The iHFLS formed were observed using a microscope on days 2 and 4 of incubation, and the polar length growth of the keratinocyte layer in the iHFLS was measured to confirm the effect of the hair-promoting agent. Minoxidil, tofacitinib, and ascorbic acid (all from Sigma–Aldrich, Saint Louis, MO, USA) were diluted in each culture medium (DMEM and Williams E) to obtain varying dose concentrations.

### 2.3. Immunostaining

Spheroids and iHFLS were placed in cryomolds using an embedding medium (OCT compound, Tissue-Tek; Miles, Napierville, IL, USA) at −80 °C. The block was cut into 7 μm sections and placed on glass slides. The slides were fixed in 4% paraformaldehyde containing 0.1% Triton X-100 for 10 min (Yakuri Pure Chemicals Co., Ltd., Osaka, Japan). The slides were blocked with bovine serum albumin (GenDEPOT, Katy, TX, USA) and incubated with anti-K14 primary antibody (Santa Cruz Biotechnology, Dallas, TX, USA) at 4 °C overnight, and then with secondary antibody (Molecular Probes, Eugene, OR, USA). Subsequently, the slides were counterstained with DAPI for 10 min. Normal mouse IgG was used as a negative control for immunostaining (R&D Systems, Minneapolis, MN, USA). Human hair follicles were embedded in cryomold and stained by the same method to compare biomarker expression with that of in vivo hair follicles.

### 2.4. Cell Tracker Staining

Cell tracker CM-DiI (Invitrogen, Burlington, ON, Canada) was attached to SV40T-hTERT DPc by the manufacturer-recommended procedure. Briefly, 2D-cultured SV40T-hTERT DPc were washed to HBSS and incubated for 30 min at 37 °C with 5% CO_2_ in CM-DiI diluted HBSS (Gibco, Burlington, ON, Canada). Subsequently, the iHFLS were developed as described earlier using CM-DiI-attached SV40T-hTERT DPc.

### 2.5. Statistical Analysis

The length of the iHFLS keratinocyte layer and the spheroid diameter visualized under the microscope were quantified using the ImageJ software (National Institute of Mental Health, Bethesda, MD, USA). The obtained lengths are represented as mean ± standard deviation (SD). The differences in the elongation between the groups were statistically analyzed using the Student’s *t*-test and one-way ANOVA using GraphPad Prism 7 software v. 7.04 (GraphPad Software Inc., La Jolla, CA, USA). *p* values < 0.05 were considered statistically significant.

## 3. Results

### 3.1. Formation of SV40T-hTERT DPc Spheroid

**Figure 2:**
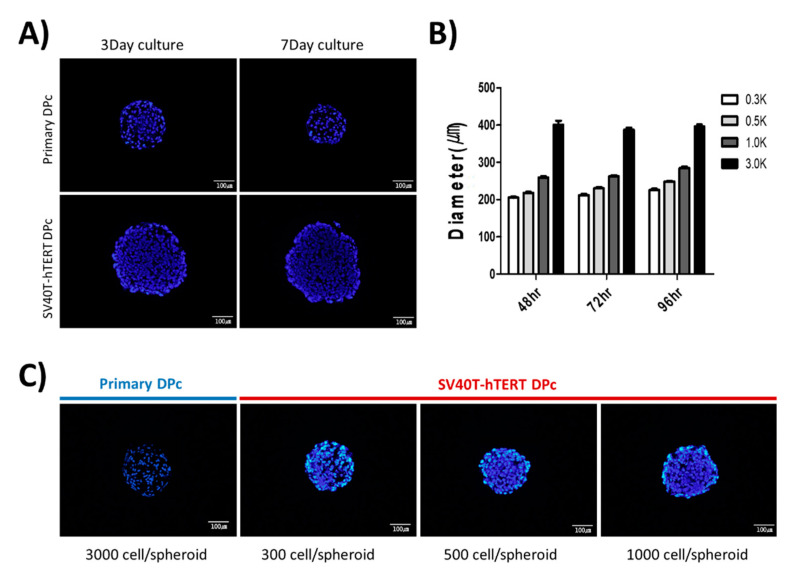
Spheroid aggregation pattern of human dermal papilla-derived cells. (**A**) Spheroid sections of primary DPc and SV40T-hTERT DPc using the same cell number (3000) for aggregation at two time points. (**B**) SV40T-hTERT DPc spheroid diameter changes over time for the initial cell population of each spheroid. (**C**) Comparison of SV40T-hTERT DPc spheroids in under 1000-cell populations with a primary DPc spheroid aggregated within 3000 cells after 72 h incubation. The cell density in the SV40T-hTERT DPc spheroids is higher than that of the primary DPc; however, the diameter of both types of spheroids is approximately the same. All sections were stained using DAPI.

The size of the dermal papilla in the human hair follicle is approximately 100–250 µm [18]. In this study, to form a dermal papilla spheroid with a similar diameter of approximately 250 µm, we observed the spheroids by monitoring the microscopic images and staining cryosections with DAPI. We confirmed that seeding approximately 3000 primary-cultured DPc into each well of a 96-well U-bottom plate resulted in spheroids with a diameter of approximately 250 µm; however, the size of the spheroid decreased slightly over time. In contrast, 3000 SV40T-hTERT DPc seedings per well of a 96-well U-bottom plate resulted in spheroid diameters higher than that observed for primary DPc (Figure 2A). Seeding 1000 SV40T-hTERT DPc resulted in an aggregated spheroid diameter of approximately 250 µm, which is comparable to the dermal papillary tissue in an anagen hair follicle. The spheroid growth pattern increased slightly; however, not significantly in diameter (Figure 2B). We confirmed that controlling the number of cells per well to <1000 resulted in the formation of immortalized SV40T-hTERT DPc spheroids with a diameter similar to that of the in vivo hair follicle (Figure 2C).

### 3.2. Optimization of the Hair Follicle-like Structure Forming Technique

To form a hair follicle-like structure, we added the hair follicle-derived keratinocytes and the outer root sheath cells (ORSc) into the spheroid-incubated U-bottom plates and compared two types of the hair follicle-like structure formation: one consisting of the primary DPc spheroid and ORSc and the other of SV40T-hTERT DPc spheroid and ORSc. These resultant hair follicle-like structure exhibited similar morphology. Therefore, we replaced primary DPc with SV40T-hTERT DPc in the subsequent hair follicle-like structure formation experiment (Video S1).

**Figure 3:**
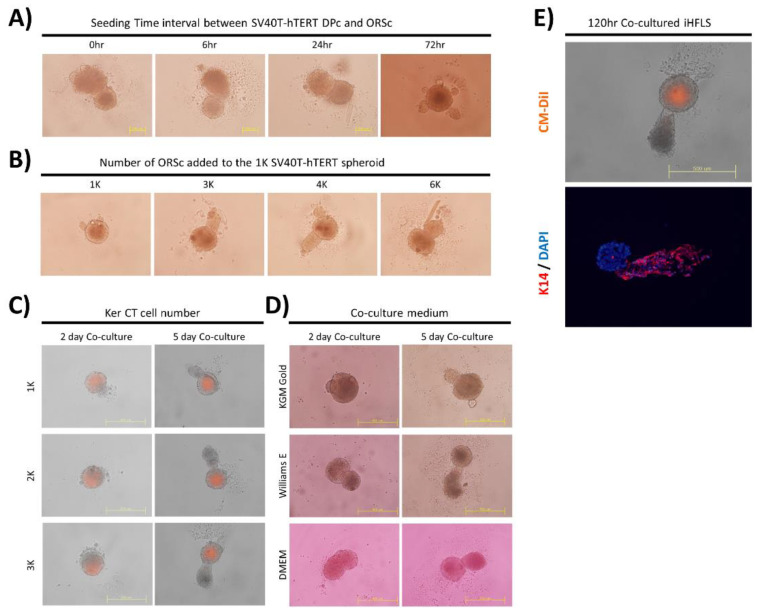
Optimal conditions for HFLS formation. (**A**) The time interval between the SV40T-hTERT DPc seeding for the spheroid formation and the start of co-culture with ORSc; approximately 1000 cells of SV40T-hTERT DPc and 3000 cells of ORSc were used. (**B**) Regulating co-cultured ORSc: Elongation of the keratinocyte layer in HFLS is prominent in the range of 3000–4000 ORSc. (**C**) Regulating immortalized keratinocyte (Ker-CT) cell numbers in HFLS composed of immortalized cells only: Addition of more keratinocytes results in larger iHFLS. (**D**) Variation of iHFLS formation according to culture medium regulation: iHFLS cultured in William’s E medium exhibits a more linear form than in other media conditions. (**E**) The cell arrangement in iHFLS using fluorescence observation: Cellular arrangement similar to hair follicles was observed using a cell tracker attached on SV40T-hTERT DPc and immunostaining of keratin 14 (K14) keratinocyte biomarker.

We observed the substantial formation of hair follicle-like structure when the ORSc was added within 24 h after the seeding of the SV40T-hTERT DPc (Figure 3A). The growth of the keratinocyte layer could be regulated in a form similar to that of hair follicles when an amount of 3000–4000 keratinocytes was added for each SV40T-hTERT DPc spheroid of 1000 cells (Figure 3B).

To form a hair follicle-like structure consisting only of immortalized cells, a hair follicle-like structure formation test was performed using an SV40T-hTERT DPc spheroid and the immortalized human neonatal foreskin cell line Ker-CT. We confirmed that the formation of an iHFLS is morphologically similar to that of the structure using primary hair follicle-derived cells. Because the immortalized cell line Ker-CT grows faster than primary ORSc, we regulated the added different Ker-CT cell numbers in the iHFLS formation progress. The results revealed that a larger number of cells resulted in the formation of a thicker structure (Figure 3C). Morphologically, the structure was mostly hair follicle-like and exhibited the longest keratinocyte layer elongation in the William’s E medium compared with that in the other media (Figure 3D).

Fluorescence imaging of the iHFLS formed by attaching the cell tracker CM-DiI to SV40T-hTERT DPc revealed that the HFLS have a similar cell arrangement structure to that of the dermal papillae located in the in vivo hair follicle. We immunostained iHFLS sections to identify the cellular composition of the elongated part in the iHFLS. The immunohistochemical staining of iHFLS sections cultured for 120 h suggested that hair follicle keratinocyte-specific marker K14 appeared in the elongated part of the hair follicle structure. These results confirmed that iHFLS mimic hair follicle morphology by combining two types of immortalized cells (Figure 3E).

### 3.3. Facilitation of iHFLS Elongation by Treating with Hair-Promoting Agent

**Figure 4:**
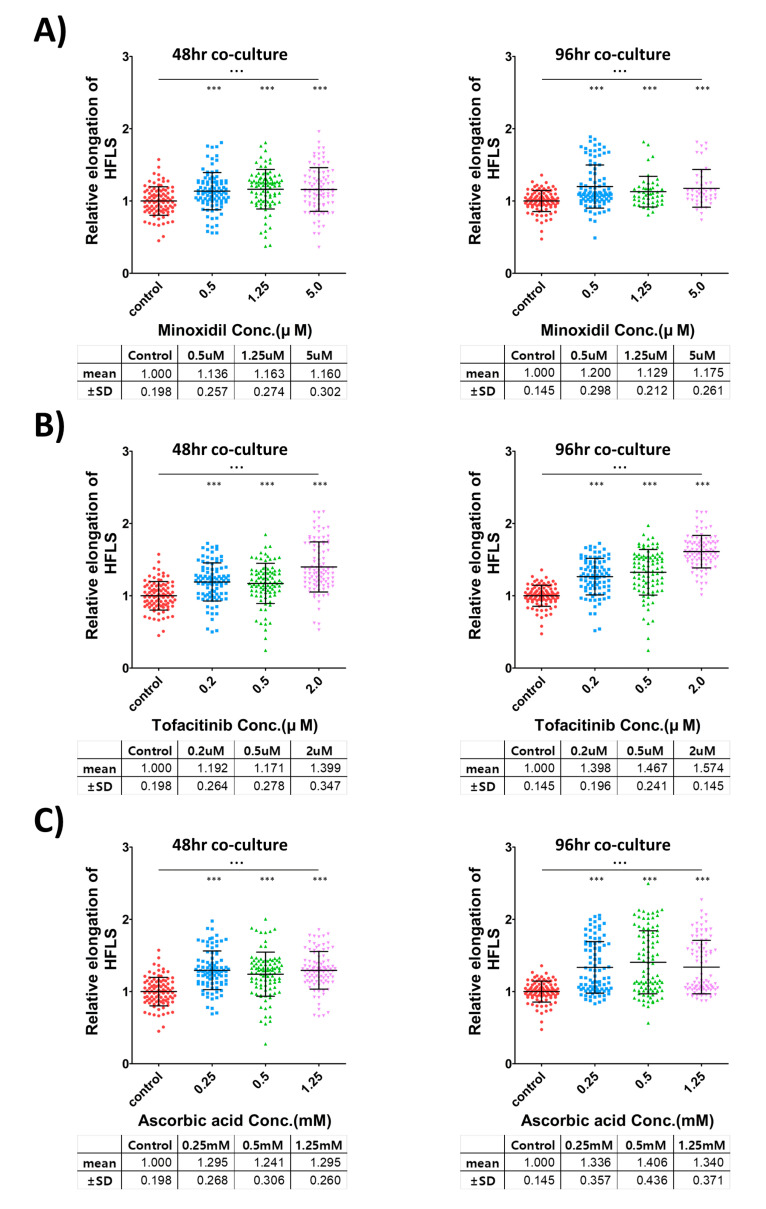
Validation of three known hair-promoting agents using iHFLS. (**A**) Relative iHFLS elongation measurement in the minoxidil treatment groups. (**B**) Relative iHFLS elongation measurement in the tofacitinib treatment groups. (**C**) Relative iHFLS elongation measurement in the ascorbic acid treatment groups. Every group showed statistically significant increased elongation compared with the control groups. *** = *p* < 0.0001 in the *t*-test, ∙∙∙ = *p* < 0.0001 in the one-way ANOVA analysis. The different color represents different concentration of treated agents.

To validate the growth-promotion effect of hair follicle growth-promoting substances in hair follicle-like structure, three kinds of reported hair follicle growth-promoting substances (minoxidil ([6]; Figure 4A), tofacitinib ([7]; Figure 4B), and ascorbic acid ([8]; Figure 4C)) were applied to iHFLS during the formation process and culture period. We confirmed the enhanced elongation of the keratinocyte layer in hair-promoting agent-treated iHFLS in a dose-related manner, with an increased minimum of 12.9% in the minoxidil 1.25 μM-treated group and a maximum of 57.4% in the tofacitinib 2 μM-treated group.

According to the Student’s *t*-test, all the hair growth-treated groups exhibited significant differences compared with that in the control group. An ANOVA analysis suggested that each concentration-specific treatment group had a significant difference. The number of iHFLS in each group was 46–98 individual structures, obtained from two independent experiments. The iHFLS length data were normalized to the average of the control group in each experiment.

## 4. Discussion

Various methods of using hair follicle tissues and hair follicle cells have previously been reported to screen treatment candidates for hair follicular growth [13,15]. However, the supply of human hair follicle tissues for research, mainly obtained from residual hair follicles after autologous hair transplantation, is insufficient to meet the demand for screening hair-promoting candidates. Potential methods have been reported using 3D-cultured hair follicle-like structure to closely mimic in vivo conditions [16]. However, in the case of methods using primary hair follicle cells, the unique characteristics of the cells are lost during 2D culture to secure the number of cells required for the experiment. These limitations and challenges in securing a sufficient number of hair follicle cells preclude the number of experiments that can be conducted. Despite the requirement for hair-promoting agents in various fields, such as pharmaceuticals, functional foods, and cosmetics, the difficulty of applying efficient screening methods hinders the development of hair-promoting agents. Therefore, the iHFLS presented in this study, which is generated using immortalized cells, can be used as a new alternative screening test model. Compared with primary DPc, the SV40T-hTERT DPc used in this study exhibited improved cell density in spheroids during 3D culture, and a larger spheroid size for the same cell number.

We confirmed that the hair follicle-like structure could be generated by a combination of keratinocytes and SV40T-hTERT DPc spheroids, which condensed into a size similar to that of in vivo dermal papilla. Hair growth varies depending on the dermal papilla size and the number of cells in the dermal papilla. Cells in the hair follicles, and the size of the in vivo dermal papilla, are variable according to the hair cycle [19,20]. These observations indicate that the cells constituting the spheroids should be controlled with suitable cell numbers to aggregate spheroids of similar size to in vivo hair follicle dermal papilla in the anagen phase when forming HFLS using immortalized cells. Additionally, our data revealed that the number of cells required to form a specific spheroid size depends on the cell type.

The growth efficiency of iHFLS keratinocyte layers is highest in the tissue culture medium compared with that in the DMEM or keratinocyte culture media. These observations suggest that balanced nutrition is more effective in the growth of iHFLS than that particularly suitable for certain types of cells. In subsequent studies, follicle-like structure growth may be controlled by adding known follicle growth-related substances to the culture medium.

The immortalized keratinocytes used in this study are cells derived from human neonatal foreskin. Therefore, the iHFLS created in this study are arguable in that they are constructed from entirely hair follicle-derived cells. However, in a recently reported study, a mimicked human hair follicle structure was grown in hydrogel using human foreskin keratinocytes [21]. Another hair study demonstrated a chimeric hair-like structure grown using human neonatal foreskin-derived cells and murine dermal cells [22]. In addition, our results show morphologically similar structure between ORSc and Ker-CT. Therefore, immortalized dermal papilla and neonatal foreskin keratinocytes could be effectively used to construct iHFLS. In future studies, immortalization of hair follicle keratinocytes could make iHFLS more similar to in vivo hair follicles and thus would be widely acceptable in disease models and validation models.

Recently, various studies have been published on the form of simulating hair follicles in an in vitro environment. Many studies on hair follicle structure neogenesis, including this study, have applied methods using the multicellular, multilayer forms of dermal papilla cell spheroids and keratinocytes. This method has the advantage of process simplicity, which benefits studies screening potential hair-promoting agents from a large compound pool.

Furthermore, studies mimicking hair follicles using various techniques have been reported. The common goal of these studies is to create an environment similar to in vivo conditions around hair follicles through various techniques, such as regulating the cell combination [23], making an in vivo skin-like structure [24], and using other bioactive substances [25]. Therefore, according to the basic experimental framework of this study, which is represented as a spheroid-keratinocyte 3D co-cultured platform, regulating the in vitro microenvironment by treatment with hormones or ECM aids in resembling hair follicles in vivo and enables the formation of human hair-follicle structure while maintaining the advantages of the simple experimental process. Furthermore, our model can be applied to the development of disease models and screening test methods. We believe that our novel model would meet the growing demand for the screening and validation of various potential candidates related to hair-care research.

## Data Availability

Not applicable.

## Supplementary Materials

The following supporting information can be downloaded at: https://www.mdpi.com/article/10.3390/bioengineering9110645/s1, Video S1: ORSc added hair follicle-like structure formation according to spheroid cell types (Primary DPc vs. SV40T-hTERT DPc).
